# Supplementary material for: Risk of metabolic syndrome in participants within the normal range of alanine aminotransferase: A population-based nationwide study
Source: PLoS One. 2020 Apr 8;15(4):e0231485. doi: 10.1371/journal.pone.0231485 (PMC7141677; doi:10.1371/journal.pone.0231485)
Supplement: S1 Table — (DOCX) [file pone.0231485.s001.docx]

**Supplementary Table 1.** Baseline characteristics of the participants

|  | Total | |  | ALT ≤40 U/L | | ALT >40 U/L | |
| --- | --- | --- | --- | --- | --- | --- | --- |
|  | Male  (n=19957) | Female  (n=26871) |  | Male  (n=17535) | Female  (n=25867) | Male  (n=2422) | Female  (n=1004) |
| Age (year, mean ± S.D.) | 49.8 ± 16.4 | 49.6 ± 16.4 |  | 50.5 ± 16.6 | 49.5 ± 16.5 | 44.7 ± 14.0 | 52.4 ± 14.3 |
| Height (cm, mean ± S.D.) | 169.6 ± 6.7 | 156.6 ± 6.6 |  | 169.5 ± 6.7 | 156.6 ± 6.6 | 170.5 ± 6.7 | 155.7 ± 6.3 |
| Weight (kg, mean ± S.D.) | 69.4 ± 11.5 | 57.4 ± 9.1 |  | 68.2 ± 10.4 | 57.1 ± 8.8 | 76.8 ± 13.3 | 63.9 ± 11.9 |
| BMI (kg/m^2^, mean ± S.D.) | 23.7 ± 2.9 | 23.4 ± 3.3 |  | 23.8 ± 3.0 | 23.3 ± 3.4 | 26.3 ± 3.7 | 26.3 ± 4.3 |
| Waist circumference (cm, mean ± S.D.) | 84.7 ± 8.9 | 78.8 ± 9.9 |  | 83.9 ± 8.6 | 78.5 ± 9.7 | 90.5 ± 9.2 | 86.7 ± 10.9 |
| SBP (mmHg, mean ± S.D.) | 121.5 ± 15.8 | 116.6 ± 18.1 |  | 121.2 ± 15.9 | 116.4 ± 18.0 | 123.3 ± 14.7 | 122.5 ± 17.5 |
| DBP (mmHg, mean ± S.D.) | 78.6 ± 10.6 | 73.8 ± 10.1 |  | 78.1 ± 10.6 | 73.6 ± 10.0 | 82.3 ± 10.6 | 77.7 ± 10.4 |
| AST (U/L, mean ± S.D.) | 24.6 ± 14.4 | 20.5 ± 9.7 |  | 22.0 ± 7.4 | 19.5 ± 5.8 | 43.5 ± 30.1 | 47.1 ± 30.7 |
| ALT (U/L, mean ± S.D.) | 26.0 ± 19.5 | 17.8 ± 13.2 |  | 20.8 ± 7.6 | 15.9 ± 6.5 | 63.6 ± 33.2 | 64.8 ± 35.8 |
| FBS (mg/dl, mean ± S.D.) | 100.9 ± 24.4 | 96.7 ± 22.2 |  | 100.2 ± 23.7 | 96.2 ± 21.2 | 106.1 ± 28.5 | 111.6 ± 36.1 |
| Total cholesterol (mg/dl, mean ± S.D.) | 187.2 ± 35.7 | 190.0 ± 36.4 |  | 185.6 ± 34.6 | 189.5 ± 36.2 | 198.8 ± 40.5 | 202.7 ± 41.0 |
| HDL cholesterol (mg/dl, mean ± S.D.) | 46.5 ± 10.9 | 51.9 ± 11.9 |  | 46.9 ± 11.0 | 52.0 ± 11.8 | 43.4 ± 9.9 | 48.1 ± 12.1 |
| Triglyceride (mg/dl, mean ± S.D.) | 158.6 ± 131.8 | 116.7 ± 80.8 |  | 148.8 ± 116.9 | 114.8 ± 77.4 | 230.1 ± 196.2 | 166.4 ± 133.4 |
| BUN (mg/dl, mean ± S.D.) | 15.3 ± 4.5 | 13.9 ± 4.4 |  | 15.4 ± 4.6 | 13.8 ± 4.4 | 14.6 ± 4.1 | 14.2 ± 4.3 |
| Creatinine (mg/dl, mean ± S.D.) | 1.0 ± 0.3 | 0.7 ± 0.2 |  | 1.0 ± 0.3 | 0.7 ± 0.2 | 1.0 ± 0.3 | 0.7 ± 0.1 |
| High risk alcohol consumption (N, mean ± S.D.) | 3957 (19.8%) | 1130 (4.2%) |  | 3313 (18.9%) | 1087 (4.2%) | 644 (26.6%) | 43 (4.3%) |
| Diabetes mellitus (N, %) | 1792 (9.0%) | 1908 (7.1%) |  | 1558 (8.9%) | 1708 (6.6%) | 252 (10.4%) | 167 (16.6%) |
| Metabolic syndrome (N, %) | 5542 (27.8%) | 7389 (27.5%) |  | 4312 (24.6%) | 6809 (26.3%) | 1230 (50.8%) | 580 (57.8%) |
| Scores of metabolic syndrome  0/1/2/3/4/5 (N) | 4594/5040/4781/3393/1754/395 | 6950/6911/5621/4117/2503/769 |  | 4421/4626/4176/2734/1309/269 | 6873/6780/5405/3853/2270/686 | 173/414/605/659/445/126 | 77/131/216/264/233/83 |

^*^Abbreviations: S.D., standard deviation; BMI, body mass index; SBP, systolic blood pressure; DBP, diastolic blood pressure; AST, aspartate aminotransferase; ALT, alanine aminotransferase; FBS, fasting blood sugar; HDL, high density lipoprotein; BUN, blood urea nitrogen.
