# Supplementary material for: Risk of metabolic syndrome in participants within the normal range of alanine aminotransferase: A population-based nationwide study
Source: PLoS One. 2020 Apr 8;15(4):e0231485. doi: 10.1371/journal.pone.0231485 (PMC7141677; doi:10.1371/journal.pone.0231485)
Supplement: S2 Table — (DOCX) [file pone.0231485.s002.docx]

**Supplementary Table 2.** Clinical characteristics of the participants according to ALT level

|  |  | Male (n=17535) | | | | |  | Female (N=25867) | | | |
| --- | --- | --- | --- | --- | --- | --- | --- | --- | --- | --- | --- |
|  | <15 U/L | 15~30 U/L | 30~40 U/L | >40 U/L | *P*-value  for trend |  | <10 U/L | 10~20 U/L | 20~40 U/L | >40 U/L | *P*-value  for trend |
| Number of participants (No, %) | n=3927 | n=10916 | n=2692 | n=2422 |  |  | n=3193 | n=16601 | n=6073 | n=1004 |  |
| Age (year, mean ± S.D.) | 50.2 ± 19.5 | 51.1 ± 15.9 | 48.4 ± 14.7 | 44.7 ± 14.0 | <0.001 |  | 37.6 ± 15.1 | 49.9 ± 16.4 | 54.8 ± 14.2 | 52.4 ± 14.3 | <0.001 |
| BMI (kg/m^2^, mean ± S.D.) | 22.3 ± 2.7 | 23.9 ± 2.9 | 25.3 ± 3.0 | 26.3 ± 3.7 | <0.001 |  | 21.4 ± 2.8 | 23.1 ± 3.2 | 24.9 ± 3.6 | 26.3 ± 4.3 | <0.001 |
| Waist circumference (cm, mean ± S.D.) | 79.9 ± 8.3 | 84.4 ± 8.2 | 88.0 ± 8.1 | 90.5 ± 9.2 | <0.001 |  | 72.9 ± 8.2 | 77.8 ± 9.2 | 83.2 ± 9.8 | 86.7 ± 10.9 | <0.001 |
| SBP (mmHg, mean ± S.D.) | 119.0 ± 16.5 | 121.6 ± 15.7 | 122.8 ± 15.3 | 123.3 ± 14.7 | <0.001 |  | 107.6 ± 14.5 | 116.2 ± 17.9 | 121.7 ± 18.0 | 122.5 ± 17.5 | <0.001 |
| DBP (mmHg, mean ± S.D.) | 75.2 ± 10.3 | 78.5 ± 10.3 | 80.8 ± 10.8 | 82.3 ± 10.6 | <0.001 |  | 69.8 ± 9.1 | 73.4 ± 9.9 | 76.1 ± 10.1 | 77.7 ± 10.4 | <0.001 |
| AST (U/L, mean ± S.D.) | 17.7 ± 4.1 | 22.1 ± 6.0 | 28.3 ± 10.8 | 43.5 ± 30.1 | <0.001 |  | 14.8 ± 5.4 | 18.4 ± 3.9 | 24.7 ± 6.6 | 47.1 ± 30.7 | <0.001 |
| ALT (U/L, mean ± S.D.) | 11.8 ± 2.0 | 20.7 ± 4.1 | 34.1 ± 3.1 | 63.6 ± 33.2 | <0.001 |  | 8.0 ± 1.2 | 14.0 ± 2.7 | 25.5 ± 5.1 | 64.8 ± 35.8 | <0.001 |
| FBS (mg/dl, mean ± S.D.) | 97.1 ± 22.9 | 100.4 ± 23.2 | 103.6 ± 26.0 | 106.1 ± 28.5 | <0.001 |  | 90.1 ± 15.0 | 95.2 ± 19.7 | 102.0 ± 26.2 | 111.6 ± 36.1 | <0.001 |
| Total cholesterol (mg/dl, mean ± S.D.) | 176.6 ± 32.2 | 187.0 ± 34.1 | 192.9 ± 37.7 | 198.8 ± 40.5 | <0.001 |  | 176.4 ± 32.7 | 189.3 ± 35.2 | 197.0 ± 38.5 | 202.7 ± 41.0 | <0.001 |
| HDL cholesterol (mg/dl, mean ± S.D.) | 48.4 ± 11.2 | 46.7 ± 10.9 | 45.2 ± 10.9 | 43.4 ± 9.9 | <0.001 |  | 54.9 ± 11.6 | 52.3 ± 11.8 | 49.6 ± 11.6 | 48.1 ± 12.1 | <0.001 |
| Triglyceride (mg/dl, mean ± S.D.) | 112.0 ± 75.1 | 151.2 ± 117.6 | 192.6 ± 144.6 | 230.1 ± 196.2 | <0.001 |  | 84.7 ± 48.8 | 109.7 ± 70.3 | 144.7 ± 96.6 | 166.4 ± 133.4 | <0.001 |
| High risk alcohol consumption (N, %) | 534 (13.6%) | 2121 (19.4%) | 658 (24.4%) | 644 (26.6%) | <0.001 |  | 165 (5.2%) | 705 (4.2%) | 217 (3.6%) | 43 (4.3%) | 0.004 |
| Diabetes mellitus (N, %) | 332 (8.5%) | 936 (8.6%) | 288 (10.7%) | 252 (10.4%) | <0.001 |  | 66 (2.1%) | 943 (5.7%) | 699 (11.5%) | 167 (16.6%) | <0.001 |
| Metabolic syndrome (N, %) | 495 (12.6%) | 2748 (25.2%) | 1069 (39.7%) | 1230 (50.8%) | <0.001 |  | 231 (7.2%) | 3862 (23.3%) | 2716 (44.7%) | 580 (57.8%) | <0.001 |

^*^Abbreviations: S.D., standard deviation; BMI, body mass index; SBP, systolic blood pressure; DBP, diastolic blood pressure; AST, aspartate aminotransferase; ALT, alanine aminotransferase; FBS, fasting blood sugar; HDL, high density lipoprotein.
