# Supplementary material for: Risk of metabolic syndrome in participants within the normal range of alanine aminotransferase: A population-based nationwide study
Source: PLoS One. 2020 Apr 8;15(4):e0231485. doi: 10.1371/journal.pone.0231485 (PMC7141677; doi:10.1371/journal.pone.0231485)
Supplement: S3 Table — (DOCX) [file pone.0231485.s003.docx]

**Supplementary Table 3.** Risk of metabolic syndrome according to serum ALT level

| Risk of metabolic syndrome | Male | | | | | | |  | Female | | | | |  |  |
| --- | --- | --- | --- | --- | --- | --- | --- | --- | --- | --- | --- | --- | --- | --- | --- |
|  | <15 U/L | 15~30 U/L | | 30~40 U/L | | >40 U/L | |  | <10 U/L | 10~20 U/L | | 20~40 U/L | | >40 U/L | |
|  | OR | OR | P-value | OR | P-value | OR | P-value |  | OR | OR | P-value | OR | P-value | OR | P-value |
| Unadjusted | 1 | 2.33 (2.10-2.59) | <0.001 | 4.57 (4.04-5.16) | <0.001 | 7.15 (6.32-8.09) | <0.001 |  | 1 | 3.89 (3.38-4.47) | <0.001 | 10.37 (8.99-11.97) | <0.001 | 17.54 (14.60-21.07) | <0.001 |
| Model 1 | 1 | 2.41 (2.17-2.68) | <0.001 | 5.24 (4.62-5.93) | <0.001 | 9.26 (8.14-10.53) | <0.001 |  | 1 | 2.21 (1.91-2.57) | <0.001 | 5.31 (4.55-6.18) | <0.001 | 11.34 (9.32-13.80) | <0.001 |
| Model 2 | 1 | 2.36 (2.12-2.62) | <0.001 | 5.05 (4.45-5.73) | <0.001 | 8.95 (7.87-10.19) | <0.001 |  | 1 | 2.21 (1.91-2.57) | <0.001 | 5.30 (4.55-6.18) | <0.001 | 11.32 (9.30-13.78) | <0.001 |
| Model 3 | 1 | 1.57 (1.40-1.76) | <0.001 | 2.54 (2.21-2.92) | <0.001 | 3.63 (3.14-4.19) | <0.001 |  | 1 | 1.59 (1.36-1.87) | <0.001 | 2.69 (2.28-3.18) | <0.001 | 4.28 (3.46-5.31) | <0.001 |

Abbreviations: ALT, alanine transaminase; OR, odds ratio

Adjusted ORs were calculated in models 1, 2, and 3. The variables for adjustment were age in model 1, age and alcohol consumption in model 2, and age, alcohol consumption, and body mass index in model 3.
