# Supplementary material for: Risk of metabolic syndrome in participants within the normal range of alanine aminotransferase: A population-based nationwide study
Source: PLoS One. 2020 Apr 8;15(4):e0231485. doi: 10.1371/journal.pone.0231485 (PMC7141677; doi:10.1371/journal.pone.0231485)
Supplement: Attachment — Submitted filename: Response to reviewers.docx [file pone.0231485.s004.docx]

Reviewer #1: The community-based study included 43402 adults with ALT<=40 U/L to investigate the associations between serum ALT levels and metabolic syndrome (MS). They found that the prevalence of MS increased with serum ALT levels. In addition, the probability of MS increased with serum ALT levels with dose-response trends. The authors concluded that ALT could be used as a marker to evaluate MS, particularly in a general population with normal ALT levels.
The work is clear and straightforward. However, there are still some comments:
1. Although the work is interesting, the clinical utility is limited. There is already clear definitions for MS as the authors indicated in the methods (MS was defined by the updated National Cholesterol Education Program Adult Treatment Panel III standards). Are there any benefits by considering ALT? By including ALT, is it superior to the original definition?

: We appreciate your valuable comment. The aim of the study was to investigate the association between serum ALT and MS. The results of our study cannot imply that the addition of ALT in the diagnostic criteria of MS would be superior to the current standards. However, ALT is a basic laboratory test done frequently in the daily clinic and increases in ALT can be a useful marker that implies the presence of MS.

2. It will be interesting to know whether serum ALT levels was correlated to any inflammatory markers or not.

: Thank you for your comment. Unfortunately, the present study did not check inflammatory markers such as tumor necrosis factor-alpha (TNF-α), plasminogen activator inhibitor 1 (PAI-1), interleukin-6 (IL-6), leptin or adiponectin. We added this limitation in the discussion section of the revised manuscript (page 14).

- Finally, the present study did not clarify the association between serum ALT level and the inflammatory markers such as tumor necrosis factor-alpha (TNF-α), plasminogen activator inhibitor 1 (PAI-1), interleukin-6 (IL-6), leptin and adiponectin.

3. It is suggested to calculate fatty liver index and examine the associations between ALT and FLI.
: We appreciate your valuable comment. As you commented, we newly analyzed the relationship between serum ALT and fatty liver. However, we could not calculate the fatty liver index (FLI) because our data did not include γ-glutamyl-transferase. Fatty liver index consists of triglyceride, BMI, γ-glutamyl-transferase and waist circumference. Therefore, we calculated the hepatic steatosis index (HSI) instead of the fatty liver index (FLI). HSI consists of ALT, AST, BMI, diabetes, and sex (Dig Liver Dis 2010;42:503-8.). We analyzed the association between ALT level and HSI. There was a dose-dependent increase of HSI as ALT levels increased in both male and female subjects (Table 2). In male subjects, HSI was 28.0 ± 3.2, 31.9 ± 3.8 and 35.9 ± 4.3 in ALT 0-15 U/L, 15-30 U/L, and 30-40 U/L, respectively (p<0.001). In female subjects, HSI was 28.0 ± 3.0, 31.4 ± 3.8 and 35.7 ± 4.4 in ALT <10 U/L, 10-20 U/L, 20-40 U/L and >40 U/L, respectively (p<0.001). We added these findings in the method section and results section of the revised manuscript (page 7 & 8 and Table 2).

- “Hepatic steatosis index (HSI) was calculated to assess the relationship between serum ALT level and fatty liver.” (page 7)
- “The mean level of HSI in male and female subjects was 31.6 ± 4.5 and 32.0 ± 4.5, respectively.” (page 8)
- There were dose-dependent increases of BMI, waist circumference, SBP, DBP, fasting blood glucose (FBS), total cholesterol, triglyceride, HSI, the prevalence of diabetes mellitus, and the prevalence of MS as ALT level increased in both male and female subjects.

Reviewer #2: The authors aimed to investigate the risk of metabolic syndrome (MS) in subjects with normal ALT level in the general population. They concluded that despite within the normal range of ALT, the risk of MS increased as the ALT levels increased. In general, the topic of this work is interesting. However, some issues should be further reconsidered or corrected.

1. The authors should provide the complete data of metabolic syndrome including systolic and diastolic blood pressure. Also, the correlation of each component of metabolic syndrome with serum ALT level should be evaluated.

: According to your comments, we added the data for systolic and diastolic blood pressure in the results section and Table 1 & 2 of the revised manuscript (page 8)

- “Systolic blood pressure (SBP) of male and female subjects was 121.2 ± 15.9 and 116.4 ± 18.0, respectively. Diastolic blood pressure (DBP) of male and female subjects was 78.1 ± 10.6 and 73.6 ± 10.0, respectively.”

Also, we analyzed the correlation of each component of metabolic syndrome with the serum ALT level (Table 4). We added these results in table 4 of the revised manuscript (page 9-10).

- “The relationship between serum ALT level and each component of MS in subjects with a normal range of ALT are shown in Table 4. In male subjects, serum ALT level were significantly associated with waist circumference (*r*=0.321, *p*<0.001), SBP (*r*=0.080, *p*<0.001), DBP (*r*=0.184, *p*<0.001), FBS (*r*=0.094, *p*<0.001), and triglyceride (*r*=0.234, *p*<0.001). In female subjects, serum ALT level were significantly associated with waist circumference (*r*=0.343, *p*<0.001), SBP (*r*=0.227, *p*<0.001), DBP (*r*=0.185, *p*<0.001), FBS (*r*=0.195, *p*<0.001), and triglyceride (*r*=0.269, *p*<0.001). There was an inverse correlation between HDL cholesterol and ALT activity in both male (*r*=-0.098, *p*<0.001) and female (*r*=-0.140, *p*<0.001).”

2. The subjects with elevated ALT levels should not be excluded and could serve as positive controls.

: According to your comments, we added the data for the subjects with elevated ALT levels in the method section, results section, and supplementary document of the revised manuscript (page 6).

- “Finally, 43,402 subjects within the normal range of alanine aminotransferase (ALT ≤40 U/L) were included in the final analysis and we compared these subjects to 3,426 subjects with ALT level >40 U/L (Figure 1).”

We also compared the risk of metabolic syndrome the subjects with normal range and abnormal range of ALT (page 11-12).

- “Clinical characteristics of the subjects with an abnormal range of ALT (>40 U/L) are presented in Supplementary table 1 and 2. In male subjects, the prevalence of MS was 12.6%, 25.2%, 39.7% and 50.8% in ALT <15 U/L, 15-30 U/L, 30-40 U/L and >40 U/L, respectively. There was a dose-dependent relationship between MS and ALT level in male subjects (*p*<0.001). In female subjects, the prevalence of MS was 7.2%, 23.3%, 44.7% and 57.8% in ALT <10 U/L, 10-20 U/L, 20-40 U/L and >40 U/L, respectively. There was a dose-dependent relationship between MS and ALT level in female subjects (*p*<0.001). We compared the risk of MS between the subjects with normal range and abnormal range of ALT (Supplementary table 3). The risk of MS in male subjects is increased when in ALT 15-30 U/L, 30-40 U/L, and >40 U/L compared to ALT<15 U/L, respectively in adjusting age, alcohol consumption, and BMI (aOR 1.57 with 95% CI: 1.40-1.76, aOR 2.54 with 95% CI: 2.21-2.92 and aOR 3.63 with 95% CI: 3.14-4.19, respectively). The risk of MS in female subjects is increased when in ALT 10-20 U/L, 20-40 U/L, and >40 U/L compared to ALT<10 U/L, respectively in adjusting age, alcohol consumption, and BMI (aOR 1.59 with 95% CI: 1.36-1.87, aOR 2.69 with 95% CI: 2.28-3.18 and aOR 4.28 with 95% CI: 3.46-5.31, respectively).”

3. The information of medication history including anti-diabetics, statin and anti-hypertensive drugs should be provided.

: As you commented, we added the information regarding the use of anti-diabetic agents, lipid lowering agents, and anti-hypertensive agents. The proportion of the usage of anti-diabetic agents in male and female subjects was 7.7% and 6.0%, respectively. The proportion of the usage of lipid lowering agents in male and female subjects was 4.7% and 5.6%, respectively. The proportion of the usage of anti-hypertensive agents in male and female subjects was 18.8% and 18.8%, respectively. We added these results in Table 1 of the revised manuscript.

4. It is noted that the manuscript is filled with some grammatical errors that need to be corrected.

: As you have commented, the manuscript has been edited for grammatical errors.
