# Supplementary material for: Risk of metabolic syndrome in participants within the normal range of alanine aminotransferase: A population-based nationwide study
Source: PLoS One. 2020 Apr 8;15(4):e0231485. doi: 10.1371/journal.pone.0231485 (PMC7141677; doi:10.1371/journal.pone.0231485)
Supplement: Attachment — Submitted filename: Response to reviewers.docx [file pone.0231485.s005.docx]

Editor’s comment: The English should be revised by native English speakers.

: We appreciate your valuable comment. According to your comment, the revised manuscript was examined by an English editing service ([www.editage.co.kr](http://www.editage.co.kr)).

Reviewer #1: all comments have been addressed adequately. It is suggested that the article could be polished by an English editor before formal publication.

: We appreciate your valuable comment. According to your comment, the revised manuscript was examined by an English editing service ([www.editage.co.kr](http://www.editage.co.kr)).

Reviewer #2: The authors aimed to investigate the risk of metabolic syndrome (MS) in subjects with normal ALT level in the general population. In general, this is an interesting topic and a clearly written paper. Also, the authors have well responded to the reviewer’s suggestions.

: We appreciate your valuable comment.
